# Association Between Patent Ductus Arteriosus and the Development of Treatment-Requiring Retinopathy of Prematurity in Preterm Infants: A Single-Center Cohort Study

**DOI:** 10.3390/children12060755

**Published:** 2025-06-11

**Authors:** Sezgin Gunes, Suzan Sahin, Ceren Durmaz Engin, Fırat Ergin, Alev Aldemir Sonmez, Özlem Bozkurt, Mehmet Yekta Oncel

**Affiliations:** 1 Division of Neonatology, Izmir City Hospital, 35540 Izmir, Turkey; sezgin_gunes@yahoo.com; 2 Division of Neonatology, Department of Pediatrics, Faculty of Medicine, Izmir Democracy University, 35140 Izmir, Turkey; 3 Department of Ophthalmology, Faculty of Medicine, Izmir Democracy University, 35140 Izmir, Turkey; cerendurmaz@gmail.com; 4 Van District Training and Research Hospital, 65300 Van, Turkey; drfiratergin@hotmail.com; 5 Department of Neonatology, Buca Seyfi Demirsoy Teaching and Research Hospital, 35390 Izmir, Turkey; aldemiralev@hotmail.com; 6 Division of Neonatology, Department of Pediatrics, Faculty of Medicine, Kocaeli University, 41001 Izmit, Turkey; drozlembozkurt@gmail.com; 7 Division of Neonatology, Department of Pediatrics, Faculty of Medicine, Izmir Katip Celebi University, 35620 Izmir, Turkey; dryekta@gmail.com

**Keywords:** retinopathy of prematurity, patent ductus arteriosus, preterm infants, respiratory support, severity

## Abstract

**Background/Objectives**: Retinopathy of prematurity (ROP) is a significant cause of childhood blindness, particularly among preterm infants. Patent ductus arteriosus (PDA) is commonly observed in neonates and may contribute to the development of ROP through increased oxygen delivery to the retina. However, the association between PDA and the severity of ROP remains unclear. This study aims to evaluate the relationship between PDA and the development of treatment-requiring ROP in preterm infants. **Methods**: A retrospective cohort study was conducted on preterm infants born between 2014 and 2020 at Izmir Private Medical Park Hospital. Infants with a birth weight of less than 2000 g and a gestational age of ≤36 + 6 weeks were included. Data on demographics, prematurity-related complications, PDA status, ROP severity, and treatment requirements were collected. Statistical analysis was performed using univariate and multivariate logistic regression models to identify predictors of ROP. **Results**: Of 516 infants, 328 did not have PDA, 117 had spontaneous PDA closure, and 71 required PDA treatment. Neonates requiring PDA treatment had significantly lower gestational age and birth weight, as well as longer respiratory support duration. PDA presence was associated with increased ROP incidence in univariate analysis (*p* < 0.001); however, it was not an independent predictor of treatment-requiring ROP in multivariate models. Significant predictors for treatment-requiring ROP included longer non-invasive ventilation duration (OR = 1.029) and total respiratory support (OR = 1.009). **Conclusions**: The findings of this study highlight the central role of respiratory morbidity in ROP pathogenesis and suggest that optimal respiratory management may be more critical for ROP prevention than PDA treatment alone.

## 1. Introduction

Retinopathy of prematurity (ROP) remains a prevalent complication among preterm infants and continues to represent a leading cause of preventable childhood blindness globally [1]. The incidence and severity of ROP are closely tied to the degree of prematurity, with the highest risk observed in neonates born at the lowest gestational ages and birth weights [2]. The pathogenesis of ROP involves an initial phase of retinal vascular arrest and obliteration induced by relative hyperoxia following birth. This is followed by a hypoxic phase, during which the ischemic retina stimulates the production of angiogenic factors, resulting in aberrant neovascularization at the junction of vascularized and avascular retina. In severe cases, this process may progress to fibrovascular proliferation and tractional retinal detachment [3].

Despite advancements in neonatal care, the incidence of treatment-requiring ROP remains substantial in many neonatal intensive care units, underscoring the multifactorial nature of the disease. Moreover, inter-center variability in oxygen saturation targeting practices and monitoring tools contributes to differences in ROP incidence across institutions.

Efforts to mitigate the risk of ROP have focused on regulating oxygen therapy in the early neonatal period. Clinical trials have demonstrated that maintaining lower oxygen saturation targets can reduce the incidence of ROP by limiting retinal oxygen toxicity, and this strategy has been widely adopted in neonatal intensive care units [4]. However, given the biphasic nature of the disease, the timing and degree of oxygen exposure may have differing effects on disease progression.

Emerging evidence suggests that fluctuating oxygen levels, rather than absolute values alone, may play a critical role in driving abnormal retinal angiogenesis.

Alongside oxygen management, controlling other modifiable risk factors—such as fluctuations in glucose levels and systemic instability—is critical, particularly in extremely preterm infants whose survival rates are improving due to advancements in perinatal care [2,3,4].

Patent ductus arteriosus (PDA) is another common concern in this population, with an incidence exceeding 70% in neonates born before 28 weeks’ gestation [5]. With a trend toward more expectant management strategies, spontaneous closure of the ductus in infants born before 26 weeks may be delayed for several weeks, with a median closure time reported as approximately 71 days [6]. The persistent patency of the ductus, particularly in the presence of a significant left-to-right shunt, may contribute to systemic hemodynamic changes. Specifically, preductal organs—including the retina—can be subjected to increased blood flow and oxygen delivery, potentially disrupting normal retinal vascular development [7,8,9].

The interplay between PDA-induced systemic effects and retinal vascularization dynamics remains insufficiently characterized, particularly in the context of prolonged ductal patency and evolving oxygenation strategies.

Although the mechanistic link between PDA and ROP is biologically plausible, data on this association remain limited and somewhat inconsistent. Prior retrospective studies, often constrained by small sample sizes, have suggested a potential relationship between hemodynamically significant PDA (hsPDA) and both the occurrence and severity of ROP [10,11]. For instance, Ford et al. [12] reported that infants born before 27 weeks with hsPDA had a higher risk of developing any stage of ROP, as well as a composite outcome of death or moderate-to-severe disease. However, the study was limited by sample size and exclusions [12].

Furthermore, the criteria used to define hsPDA and the timing of ROP assessment vary considerably across studies, making it challenging to draw definitive conclusions.

In the present study, we aim to evaluate the relationship between PDA—particularly its presence and hemodynamic significance—and the development of treatment-requiring ROP in a larger cohort of preterm infants in our local clinical setting. Understanding this association may offer important insights for optimizing the management of both PDA and ROP in vulnerable neonates.

## 2. Materials and Methods

Between January 2014 and 31 December 2020, the hospital records of all premature infants who were admitted to the Neonatal Intensive Care Unit of İzmir Private Medical Park Hospital and underwent ROP screening during this 7-year period were retrospectively reviewed. Data were collected on demographic characteristics, preterm birth risk factors, duration of oxygen support, imaging findings, prematurity-related complications (such as NEC, ROP, IVH), clinical and echocardiographic findings of PDA, need for and method of PDA treatment, ROP stage, need for ROP treatment, and mortality outcomes.

The inclusion criteria for the study were: being born between 2014 and 2020, having a birth weight of less than 2000 g, being born at ≤36 + 6 weeks of gestation, and having been hospitalized at the aforementioned institution until discharge. The exclusion criteria included: significant missing data in medical records, the presence of genetic or major congenital anomalies, transfer to another hospital for ongoing treatment, and term infants who had undergone ROP screening.

PDA diagnosis and description criteria were made according to the guidelines set by the Turkish Neonatal Society [13], and staging was performed based on echocardiographic parameters including ductal diameter, flow pattern, left atrial to aortic root ratio, and signs of systemic hypoperfusion. Hemodynamically significant PDA (hsPDA) was defined as a ductal diameter ≥1.5 mm with echocardiographic evidence of left heart volume overload or diastolic flow reversal in the descending aorta.

ROP staging, screening and treatment indications were concordant with the related guidelines of the Turkish Neonatal Society which is based on the International Classification of Retinopathy of Prematurity and the Early Treatment for Retinopathy of Prematurity criteria [14]. ROP severity was staged from 1 to 5 according to the International Classification of Retinopathy of Prematurity (ICROP). Stage 1 indicates a demarcation line between vascularized and avascular retina, while Stage 2 is characterized by the presence of a ridge at the demarcation line. Stage 3 involves extraretinal neovascularization extending into the vitreous, whereas Stage 4 corresponds to partial retinal detachment. Stage 5 represents the most advanced form of the disease, with total retinal detachment. Regarding anatomical localization, Zone I is defined as a circle centered on the optic disk with a radius twice the distance from the disk to the macula. Zone II extends from the periphery of Zone I to the ora serrata nasally, and Zone III comprises the residual temporal crescent of retina not included in Zone II. Furthermore, the presence of plus disease—characterized by dilated and tortuous posterior pole vessels in at least two quadrants—was assessed to determine disease severity. All diagnostic classifications were made in accordance with ICROP criteria [15].

Treatment-requiring ROP was defined in accordance with the ETROP criteria, including any Stage ROP with plus disease in Zone I, Stage 3 in Zone I with or without plus disease, and Stage 2 or 3 in Zone II with plus disease. All infants requiring ROP treatment in our cohort received laser photocoagulation as monotherapy. Intravitreal anti-vascular endothelial growth factor (VEGF) injections were not performed, as per institutional protocol. Infants with Zone I disease who were candidates for anti-VEGF therapy were referred to a tertiary university hospital for treatment and were therefore excluded from this study [16].

The first ROP examination was performed at 32 weeks of postmenstrual age for infants born at <28 weeks of gestational age, and at postnatal week 4 for those born at ≥28 weeks, in accordance with national screening guidelines. Follow-up examinations continued until complete retinal vascularization was achieved or until the infant reached 45 weeks of postmenstrual age, whichever occurred first [14].

The aim of this study was to retrospectively evaluate, in preterm infants, the relationship between the echocardiographically determined severity of the PDA shunt and the severity of ROP. This included: a comparison of ROP treatment requirements among infants with no PDA, spontaneously closed PDA, and PDA requiring medical treatment; a comparison of pregnancy- and prematurity-related complications among infants with no PDA, spontaneously closed PDA, and PDA requiring medical treatment; identification of significant risk factors for the development of any stage of ROP; and identification of significant risk factors for the development of ROP requiring treatment.

### Statistical Analysis

Statistical analysis was conducted using SPSS for Windows, version 25.0 (IBM Corp., Armonk, NY, USA). Descriptive statistics were presented as means and standard deviations for continuous variables, and as frequencies and percentages for categorical variables. For group comparisons, one-way ANOVA was used for parametric continuous variables, while the Kruskal–Wallis test was applied for non-parametric data. Categorical variables were analyzed using Pearson’s chi-square test or Fisher’s exact test, depending on expected cell counts. In the multivariate logistic regression analysis, variables with *p*-values less than 0.10 in the univariate analysis were included. The enter method was used for variable selection in the logistic regression models. Odds ratios (ORs) and 95% confidence intervals (CIs) were calculated for each variable. A *p*-value of <0.05 was considered statistically significant.

## 3. Results

Of the 516 infants analyzed in the study, 328 did not have PDA (Group 1), 117 had experienced spontaneous PDA closure (Group 2) and 71 had PDA requiring treatment (Group 3).

Neonates requiring PDA treatment (Group 3) had significantly lower gestational age (GA) (*p* < 0.001) and birth weight (BW) (*p* = 0.031) compared to those without PDA (Group 1). Additionally, APGAR 1 (*p* = 0.016) and APGAR 5 (*p* < 0.001) scores were lower in neonates with spontaneously closed PDA (Group 2) than in those without PDA. The duration of MV (*p* < 0.001), non-invasive ventilation (NIV) (*p* < 0.001), and total respiratory support (*p* < 0.001) was significantly longer in Group 2 and Group 3 compared to Group 1, highlighting the increased respiratory burden in neonates with PDA. These findings suggest that lower GA and BW, along with prolonged respiratory support, are associated with PDA persistence and treatment need. Detailed results are given in Table 1.

Maternal factors except pre-eclampsia were similar in between the groups. Neonates requiring PDA treatment (Group 3) had a significantly higher incidence of pre-eclampsia (*p* = 0.007) and respiratory distress syndrome (RDS) (*p* < 0.001) compared to the other groups. Delivery-related factors such as history of CPR (*p* = 0.016) and preterm premature rupture of membranes (PPROM) (*p* = 0.019) were also more frequent in Group 2 and Group 3. Regarding developmental factors, small for gestational age (SGA) (*p* < 0.001) and intrauterine growth restriction (IUGR) (*p* < 0.001) were more common in Group 1, whereas AGA (*p* < 0.001) was significantly higher in Group 3. Among concomitant diseases, bronchopulmonary dysplasia (BPD), pneumothorax, late sepsis, and mortality rates were significantly increased in Group 3 (*p* < 0.001). These findings suggest that neonates requiring PDA treatment present with a higher burden of respiratory complications and systemic morbidities. Detailed results are given in Table 2.

Neonates requiring treatment for PDA closure had a significantly higher incidence of ROP compared to those with spontaneous PDA closure or without PDA (*p* < 0.001). The distribution of no ROP, spontaneously regressed ROP, and treatment-needed ROP differed significantly between groups. Additionally, Spearman’s correlation analysis revealed a significant positive association between PDA status and ROP severity (*p* < 0.001). These findings indicate that PDA closure status may be linked to ROP progression, with the highest risk observed in neonates requiring PDA treatment. Detailed results are given in Table 3.

Univariate analysis showed that GA (*p* < 0.001), BW (*p* < 0.001), APGAR1 (*p* = 0.001), MV time (*p* = 0.028), NIV time (*p* < 0.001), total O2 support time (*p* < 0.001), presence of PDA (*p* < 0.001), RDS (*p* < 0.001), preeclampsia (*p* = 0.001), late neonatal sepsis (*p* = 0.010), SGA (*p* = 0.001), IUGR (*p* = 0.001), and BPD (*p* < 0.001) were associated with any stage of ROP.

Based on those findings, two distinct multivariate logistic regression models were constructed to explore different aspects of ROP risk. Model I assessed factors associated with the development of any-stage ROP (presence vs. absence), incorporating variables with *p* < 0.05 in univariate comparisons, including GA, BW, PDA status, total duration of respiratory support, and BPD. In contrast, Model II focused specifically on identifying predictors of treatment-requiring ROP versus non-treatment cases (including both no ROP and spontaneously regressed ROP). This model included variables such as GA, non-invasive ventilation duration, total respiratory support duration, and BPD as covariates, all of which were found to be statistically significant in univariate analysis.

The Model I is statistically significant, and BPD is the strongest predictor of ROP at any stage, with an odds ratio of 0.307 (*p* < 0.001), indicating that it significantly reduces the likelihood of ROP. The presence of PDA is borderline significant (*p* = 0.079), suggesting a potential but inconclusive effect. Gestational age, BW, respiratory support duration, and RDS are not statistically significant predictors in the multivariate analysis. The model accurately predicts non-ROP cases (93.8% specificity) but has low sensitivity (27.3%) in identifying actual ROP cases.

The results of Model II indicate that NIV duration (*p* = 0.017, OR = 1.029) and total respiratory support duration (*p* = 0.041, OR = 1.009) are significant predictors of treatment-needed ROP development. Each additional day of NIV increases the odds of developing ROP by 2.9%, while longer total respiratory support duration is also associated with a higher risk of ROP. Birth weight (*p* = 0.080, OR = 0.998) is borderline significant, suggesting that lower birth weight may slightly increase ROP risk, though this result is not strongly conclusive. Other variables, including GA, presence of PDA, RDS, and BPD, did not show statistically significant effects on ROP development. Specifically, PDA presence (*p* = 0.894, OR = 0.948) does not exhibit a strong relationship with ROP in this dataset, and RDS (*p* = 0.996, OR = 0.000) appears to be an outlier due to extreme Beta values, likely a result of data limitations (Table 4).

## 4. Discussion

In this large, single-center retrospective cohort study, we evaluated the association between the presence and severity of PDA and the development of treatment-requiring ROP in preterm infants. Our findings demonstrate that although PDA presence was associated with increased ROP incidence in univariate analysis, PDA itself was not an independent predictor of ROP development when controlling for key confounding factors, notably respiratory support duration and BPD.

In a prospective cohort study, which included 6115 infants with birth weight ≤1500 g, lower birth weight and/or gestational age, prolonged duration of oxygen therapy, late-onset sepsis, increased frequency of red blood cell transfusions, and accelerated relative weight gain were identified as independent risk factors for the development of severe ROP. In this study, infants with PDA and required treatment had higher rates of severe ROP compared to no ROP (53% vs. 36%; *p* < 0.001) [17]. Other previous studies have suggested a plausible pathophysiological link between PDA and ROP. The left-to-right shunting in PDA results in persistent pulmonary overcirculation and increased preductal oxygen delivery, particularly to sensitive organs such as the brain and retina [18]. Elevated partial oxygen pressures, even under conservative oxygen targeting, can exacerbate the biphasic mechanism of ROP, promoting early vasoconstriction and later neovascularization [19,20]. Retrospective cohort studies, such as that by Madan et al., have reported higher rates of ROP among infants with hemodynamically significant PDA (hsPDA), while Noori et al. emphasized that untreated PDA could prolong systemic oxygen imbalance, potentially worsening ROP outcomes [21,22]. Our findings are partially consistent with these studies in that PDA was associated with increased ROP rates; however, multivariate analysis revealed that PDA did not independently predict severe ROP requiring treatment.

Similarly, Ford et al. [23] reported that hsPDA was linked to a higher risk of any-stage ROP and the composite outcome of death or severe ROP in extremely low gestational age neonates. However, their study included a relatively small sample size (n = 86) and did not fully adjust for confounding respiratory variables [23]. In contrast, our study, with a larger sample (n = 516), incorporated detailed multivariate models accounting for respiratory support duration, providing a more nuanced understanding. We identified that the duration of respiratory support, particularly NIV and total oxygen exposure, had a stronger association with severe ROP than PDA presence itself, aligning with large-scale studies such as the SUPPORT trial, which highlighted the central role of oxygen management in ROP pathogenesis [20].

Moreover, BPD emerged as the strongest independent risk factor for ROP at any stage in our study. This association has been well documented, where prolonged oxygen supplementation and mechanical ventilation contribute both to lung injury and impaired retinal vascular development [19,24]. Indeed, Jensen et al. proposed that BPD and ROP may share common pathways involving oxidative stress, inflammation, and disrupted angiogenesis, supporting the strong predictive value of BPD observed here [24].

Interestingly, although PDA closure status was linked to increased systemic morbidity—such as higher rates of RDS, late sepsis, pneumothorax, and mortality—this overall burden of illness may act as a confounder rather than a mediator in the PDA-ROP relationship. Similar complexities were noted by Jhaveri et al., who argued that PDA is often a marker of overall immaturity and illness severity rather than a direct contributor to specific complications like ROP [25].

Our study contributes valuable evidence to this ongoing debate by emphasizing that PDA alone, particularly when managed conservatively, may not significantly drive ROP progression independently of respiratory and systemic factors. However, given that infants with persistent PDA exhibited longer durations of mechanical and non-invasive ventilation, it remains clinically prudent to monitor these neonates closely for ROP progression.

The strengths of our study include the large cohort size, detailed clinical characterization, and multivariate modeling adjusting for major known risk factors. In our cohort, all infants with treatment-requiring ROP underwent laser photocoagulation, as intravitreal anti-VEGF therapy was not utilized during the study period. This consistent treatment approach minimized variability and enabled uniform outcome assessment. Nevertheless, limitations include the retrospective design, which may lead to incomplete data capture. Another limitation of our study is the unequal distribution of participants across the groups, which may have influenced statistical power. However, appropriate statistical methods were applied to minimize potential bias related to this imbalance. Additionally, the study was conducted in a single tertiary care center, which may limit the generalizability of the findings to different populations with varying clinical practices, socioeconomic backgrounds, and PDA management approaches. Larger, multicenter prospective studies would be valuable to validate and extend these results.

Furthermore, potential treatment effects (e.g., pharmacological PDA closure) on systemic oxygenation and retinal outcomes were not separately analyzed, an area that future prospective studies could address.

Finally, studies integrating detailed oxygenation profiles, including fluctuations in oxygen delivery and cumulative oxygen exposure, may provide deeper insights into the complex interplay between PDA, respiratory support, and ROP development.

## 5. Conclusions

In this large cohort of preterm infants, we found that while the presence of PDA was associated with an increased incidence of ROP in unadjusted analyses, it did not independently predict treatment-requiring ROP when controlling for key confounding factors such as respiratory support duration and BPD. Our findings underscore the critical role of respiratory morbidity in ROP pathogenesis and suggest that targeted management strategies focused on optimizing respiratory care may have a greater impact on ROP prevention than PDA-directed interventions alone. Future prospective, multicenter studies are needed to further delineate the complex interplay between PDA, respiratory support, and ROP development in this vulnerable population.

## Figures and Tables

**Table 1:** Comparison of demographic parameters between neonates with no PDA (group 1), spontaneously closed PDA (group 2) and treatment-needed PDA (group 3).

	Group	Mean ± SD	Min–Max	*p* *	*p* **
GA, weeks	1	29.2 ± 2.5	22–36	**<0.001**	**1–2: <0.001**
	2	27.3 ± 2.3	22–33		**1–3: <0.001**
	3	27.4 ± 2.2	23–32		2–3: 1.000
BW, grams	1	1134.3 ± 265.3	410–1700	**<0.001**	**1–2: <0.001**
	2	998.6 ± 262.7	410–1550		**1–3: 0.031**
	3	1043.8 ± 291.8	540–1595		2–3: 0.790
Age of the mother	1	30.0 ± 5.9	16–43	0.695	1–2: 1.000
	2	29.5 ± 5.9	17–44		1–3: 1.000
	3	30.3 ± 6.1	17– 53		2–3: 1.000
APGAR 1	1	5.8 ± 1.6	0–9	**0.016**	**1–2: 0.014**
	2	5.3 ± 1.7	1–8		1–3: 0.817
	3	5.6 ± 1.8	1–9		2–3: 0.842
APGAR 5	1	7.5 ± 1.0	1–10	**<0.001**	**1–2: <0.001**
	2	7.1 ± 1.1	2–9		1–3: 1.000
	3	7.5 ± 0.8	4–9		**2–3: 0.033**
MV time, days	1	2.4 ± 13.6	0–166	**<0.001**	**1–2: <0.001**
	2	14.9 ± 47.1	0–255		1–3: 0.059
	3	11.8 ± 46.3	0–368		2–3: 1.000
NIV time, days	1	11.1 ± 14.4	0–72	**<0.001**	**1–2: <0.001**
	2	23.6 ± 20.8	0–106		**1–3: <0.001**
	3	21.3 ± 14.9	0–61		2–3: 1.000
Total respiratory support time, days	1	28.3 ± 28.7	0–211	**<0.001**	**1–2: <0.001**
	2	51.4 ± 49.4	2–255		**1–3: <0.001**
	3	62.0 ± 53.7	2–368		2–3: 0.216

* ANOVA; ** Post hoc Bonferroni; BW, birth weight; GA, gestational age; MV, mechanic ventilation; NIV, non-invasive ventilation.

**Table 2:** Comparison of maternal, delivery-related, developmental factors and concomitant diseases among neonates without patent ductus arteriosus (PDA) (Group 1), those with spontaneous PDA closure (Group 2), and those requiring treatment for PDA closure (Group 3).

	Group 1 (n = 328)	Group 2 (n = 117)	Group 3 (n = 71)	*p* *
***Maternal factors*** **(n,%)**				
GDM	26 (8)	6 (5)	5 (7)	0.639
Pre-eclampsia	67 (20)	9 (8)	13 (18)	**0.007**
Smoking	26 (8)	7 (6)	10 (14)	0.129
Multiple pregnancy	103 (31)	37 (31)	29 (40)	0.295
Primigravida	159 (48)	56 (49)	28 (39)	0.476
***Delivery-related factors*** **(n,%)**				
Type of delivery (C/S)	287 (87)	95 (81)	59 (83)	0.196
History of CPR	20 (6)	17 (14)	8 (11)	**0.016**
PPROM	77 (23)	43 (37)	17 (23)	**0.019**
Chorioamnionitis	8 (2)	2 (2)	4 (6)	0.267 ‡
Ablatio placenta	9 (2)	5 (4)	0 (0)	0.224 ‡
Oligohydroamnios	20 (6)	9 (8)	6 (8)	0.736
Polihydroamnios	3 (1)	0 (0)	0 (0)	0.724 ‡
***Developmental factors*** **(n,%)**				
SGA	80 (24)	10 (8)	6 (8)	**<0.001**
LGA	0 (0)	1 (1)	1 (1)	0.132 ‡
AGA	180 (54)	54 (46)	63 (88)	**<0.001**
IUGR	85 (26)	13 (11)	3 (4)	**<0.001** ‡
***Concomittant diseases*** **(n,%)**				
Congenital anomaly	64 (19)	39 (33)	3 (4)	**<0.001** ‡
RDS	179 (54)	102 (87)	59 (83)	**<0.001**
Surfactant use	124 (38)	58 (50)	56 (79)	**<0.001**
Pneumothorax	4 (1)	15 (13)	3 (4)	**<0.001** ‡
BPD	129 (39)	72 (61)	59 (83)	**<0.001**
Feeding intolerance	159 (48)	64 (55)	44 (62)	0.094
NEC	20 (6)	8 (7)	7 (10)	0.538
IVH	19 (6)	12 (10)	5 (7)	**0.015**
Early sepsis	6 (2)	5 (4)	0 (0)	0.169 ‡
Late sepsis	89 (27)	17 (14)	31 (43)	**<0.001**
Mortality	5 (1)	17 (14)	3 (4)	**<0.001** ‡

* *p* values were calculated using the χ^2^ test when appropriate and Fisher’s exact test (‡) when any expected cell frequency was <5. AGA, appropriate for gestational age; BPD, bronchopulmonary dysplasia; C/S, cesarean section; GDM, gestational diabetes mellitus; IUGR, intrauterine growth restriction; IVH, intraventricular hemorrhage; LGA, large for gestational age; NEC, necrotizing enterocolitis; PPROM, preterm premature rupture of membranes; RDS, respiratory distress syndrome; SGA, small for gestational age.

**Table 3:** Comparison of retinopathy of prematurity (ROP) status among neonates without patent ductus arteriosus (PDA) (Group 1), those with spontaneous PDA closure (Group 2), and those requiring treatment for PDA closure (Group 3).

	No ROP (n,%)	Spontanously Regressed ROP (n,%)	Treatment-Needed ROP (n,%)	*p* Value *	Spearman’s Correlation Value; *p* Value
Group 1 (n = 328)	263 (80.2%)	50 (15.2%)	15 (4.6%)	**<0.001**	0.185; **<0.001**
Group 2 (n = 117)	74 (63.2%)	26 (22.2%)	17 (14.5%)		
Group 1 (n = 328)	263 (80.2%)	50 (15.2%)	15 (4.6%)	**<0.001** ‡	0.204; **<0.001**
Group 3 (n = 71)	40 (56.3%)	27 (38.0%)	4 (5.6%)		
Group 2 (n = 117)	74 (63.2%)	26 (22.2%)	17 (14.5%)	**0.026** ‡	0.026; 0.737
Group 3 (n = 71)	40 (56.3%)	27 (38.0%)	4 (5.6%)		

* *p* values were calculated using the χ^2^ test when appropriate and Fisher’s exact test (‡) when any expected cell frequency was <5. Percentages were calculated within each group based on the group-specific total (n). ROP, retinopathy of prematurity.

**Table 4:** Multivariable logistic regression models assessing risk of any stage of ROP development or treatment-needed ROP development.

	Model I				Model II			
Variable	Odds Ratio	B (Beta)	S.E.	*p*-Value	Odds Ratio	B (Beta)	S.E.	*p*-Value
GA, weeks	0.977	−0.023	0.073	0.754	**1.052**	**0.051**	**0.125**	**0.682**
BW, grams	1.000	0.000	0.001	0.856	**0.998**	**−0.002**	**0.001**	**0.080**
NIV duration, days	1.004	0.004	0.009	0.615	1.029	**0.029**	0.012	**0.017**
Total respiratory support time, days	1.005	0.005	0.004	0.188	1.009	0.009	0.004	**0.041**
PDA (yes = 1)	0.656	−0.422	0.240	0.079	0.948	−0.054	0.403	0.894
RDS (yes = 1)	0.810	−0.210	0.305	0.490	0.000	−17.912	3295.740	0.996
BPD (yes = 1)	**0.307**	**−1.181**	**0.313**	**<0.001**	0.936	−0.066	0.622	0.916

GA, gestational age; BW, birth weight; NIV, non-invasive ventilation; PDA, patent ductus arteriosus; RDS, respiratory distress syndrome; BPD, bronchopulmonary dysplasia.

## Data Availability

Data is publicly unavailable due to privacy/ethical restrictions but may be supplied upon reasonable demand for academic research.
